# The Raison D’être for the Community Pharmacy and the Community Pharmacist in Sweden: A Qualitative Interview Study

**DOI:** 10.3390/pharmacy4010003

**Published:** 2015-12-25

**Authors:** Kristin Wisell, Sofia Kälvemark Sporrong

**Affiliations:** 1 Department of Pharmacy, Uppsala University, Box 580, S-751 23 Uppsala, Sweden; 2 Department of Pharmacy, University of Copenhagen, Universitetsparken 2, 2100 København Ø, Denmark; sofia.sporrong@sund.ku.dk

**Keywords:** community pharmacy, regulation, pharmacy policy, pharmacy reform, legislation, Sweden

## Abstract

Community pharmacies are balancing between business (selling medicines and other products) and healthcare (using the pharmacists’ knowledge in order to improve drug utilization). This balance could be affected by regulations decided upon by politicians, but also influenced by others. The aim of this study was to explore important stakeholders’ views on community pharmacy and community pharmacists in Sweden. The method used was that of semi-structured qualitative interviews. Political, professional, and patient organization representatives were interviewed. The results show that informants who are pharmacists or representatives of a professional pharmacist organization generally have a healthcare-centered view on community pharmacy/pharmacists. However, different views on how this orientation should be performed were revealed, ranging from being specialists to dealing with uncomplicated tasks. Political organization representatives generally had a more business-oriented view, where competition in the market was believed to be the main driving force for development. A third dimension in which competition was not stressed also emerged; that community pharmacies should primarily distribute medicines. This dimension was most prevalent among the political and patient organization representatives. One conclusion to be drawn is that no stakeholder seemed to have a clear vision or was willing to take the lead for the development of the community pharmacy sector.

## 1. Introduction

Are pharmacists in community pharmacies selling medicines or dispensing (preparing, checking the correctness of the prescription, ensuring that the patient will use the medicine correctly, and handing the medicine to the patient) prescriptions? There is a conflict between business and healthcare perspectives within community pharmacies. Many healthcare settings can have economic incentives, but it is said to be stronger in community pharmacies because products are sold “in a context which resembles a retail establishment more closely than it does a professional office” ([1], p. 103). Pharmacies can, on the one hand, be seen as a business based on selling a product—medicine. On the other hand, they are part of the healthcare sector and have healthcare professionals working there [2,3]. In many countries there is a trend towards integrating pharmacies and community pharmacists into the healthcare sector, e.g., by allowing pharmacists to make therapeutic substitutions, or to renew, modify, or initiate prescriptions, either independently or in collaboration with prescribers [4,5]. This trend gives more opportunities for pharmacists’ expertise to be used in patient-centered care, working towards an appropriate use of medicines [5].

Different models can be applied to the two perspectives of business and healthcare. The professional model is based on trust in the judgment of the individuals in the profession. In healthcare, professionalism has traditionally been an important value; it is motivated by the urge to do a good job in and of itself for the benefit of patients, and the will to get collegial endorsement, regardless of economic incentives. According to this view, economic incentives should rather be avoided in healthcare, as they could risk diminishing the professional motivation to care about the patient. This is because economic incentives could tend to modify the health professionals’ motivations—from being patient advocates into caring about money [6].

A market model, on the other hand, would look at community pharmacies as businesses. Hence, the activities are seen as consumers in the market making well-informed, independent decisions, e.g., based on the quality of the service provided [7]. According to this perspective, many individuals, in this case patients, can together make better-informed decisions than can a few at the top [8]. Typically, customer satisfaction is important. This kind of “consumerism” in healthcare is, however, argued to be problematic, firstly as short-term goals will be considered to a greater extent than long-term goals will, and secondly as good relations with healthcare personnel could be confused with quality of care [9].

Regulations both play an important role in the balance between the two perspectives and are also the most apparent means of changing this balance in community pharmacy. Regulations on reimbursement for pharmacies directly influence their income, and reimbursing pharmaceutical services can be one way to (try to) change behavior towards a healthcare perspective. Regulations on the establishment of pharmacies will have economic consequences, such as allowing more pharmacies to open, giving each less prescriptions to handle and, hence, less income from selling prescription medicines [10]. It has been shown that countries with relatively liberalized ownership regulations regarding community pharmacies have seen an increase in the number of pharmacies, which indicates an increased focus on profit (through more competition) [11].

The political process leading to a change of regulations is typically influenced not only by politicians, but also by other stakeholders, as well as by attitudes in society, especially within groups/organizations interested in the matter. In order to understand in which direction the roles of community pharmacies and pharmacists are developing, it is interesting to investigate the attitudes of influential key stakeholders. This is especially relevant in the view of major policy changes, such as the one in Sweden (see below) which can be interpreted as moving towards a more business-centered model [12].

The aim of this study was to explore influential stakeholders’ views on the role of the community pharmacist and community pharmacies in Sweden.

## 2. Experimental Section

### 2.1. The Setting

The Swedish state-owned community pharmacy monopoly was abolished in 2009, and two-thirds of the existing pharmacies were sold to private owners. The main purpose of the reform was to get more pharmacies and competition, and it was ideologically driven [12].

After the liberalization, no ownership or location restriction exists, and anyone (except prescribers or medicine producers) can open and own a pharmacy anywhere [13]. Before the liberalization, the Swedish pharmacy sector consisted of 930 community pharmacies [14]. Today the number is 1400 [15], primarily organized in large pharmacy chains [16,17]. About one-third of the pharmacies are still owned by the state, and 165 are owned by individuals (pharmacists or others) [16].

### 2.2. Method

The method used in this study was semi-structured qualitative interviews with key stakeholders within the Swedish pharmacy sector. Representatives from political, professional, and patient organizations were interviewed. Altogether, 12 interviews were performed by the first author between March and October 2013. The interviews lasted between 30 and 60 min, and were audio-recorded and transcribed verbatim.

An interview guide with semi-structured questions was developed. Themes in the guide included different views of the roles of the community pharmacists and pharmacies, and the effects and rationales for the ownership liberalization of the Swedish community pharmacy sector. This article deals only with the perspectives on the role of the pharmacy/pharmacist (the effects and rationales for the ownership liberalization are reported elsewhere).

The organizations were chosen with a combination of snowball and purposeful sampling [18]. A pharmacy market consultant was initially interviewed in order to identify important organizations regarding issues concerning the Swedish pharmacy sector. Also, the informants were asked to suggest further organizations to include. Each organization chose a person within their organizations whom they considered as the most knowledgeable when it comes to community pharmacists and pharmacies.

Representatives of the following organizations were interviewed:

- Political parties (national level): New Conservatives (Moderaterna), The Liberal Party of Sweden (Folkpartiet), The Centre Party (Centerpartiet), The Christian Democrats (Kristdemokraterna), The Social Democrats (Socialdemokraterna);
- The Swedish Association of Local Authorities and Regions (Sveriges Kommuner och Landsting);
- Professional organizations: The Swedish Pharmaceutical Society (Apotekarsocieteten), The Swedish Pharmaceutical Union (Farmaciförbundet), The Swedish Pharmacists (Sveriges Farmaceuter);
- Patient organizations: National Pensioners’ Organization (PRO), The Swedish Association for Senior Citizens (SPF), The Rheumatic Association (Reumatikerförbundet).

The analysis was made in several steps [19]; first, parts of the interviews dealing with the role of pharmacies and pharmacists were identified. Secondly, the duality of business and health was used as a framework for the analysis, and relevant parts were deductively put into the two categories of business and healthcare, first independently by the two authors and then as discussed in a consensus meeting. During the process, a third category emerged (distribution), which led to a second round of analysis (independent and consensus). Lastly, an inductive sub-categorization was made within the main categories.

Quotes have been chosen to illustrate the categories. Every interviewee is represented by a number (which is not in the same order as in the list of organizations). Some of the interviewees were themselves pharmacists, or representatives of a professional pharmacist organization. During the analysis, it became clear that the opinion of those that had a pharmacist education differed from the other informants. Hence, quotes coming from these categories are marked with “PH”. In the text, these interviewees are called “pharmacist/professional representatives”.

Approval from an ethics review committee was, according to Swedish regulations, not necessary. Ethical considerations were, however, met. Informants were informed about the study and told that their participation was voluntary.

## 3. Results

First, the healthcare dimension is presented. Within this dimension, different views are shown on the pharmacists’ expertise and how it should be used. After this, the business dimension is described, where customers are seen as important in developing the services performed in the pharmacies. Finally, the third dimension—distribution of the product—is presented.

### 3.1. Healthcare (Pharmacists’ Expertise in Focus)

Overall, three different perspectives were present in regard to community pharmacists as part of healthcare: (1) they have a unique expertise concerning medicines and should be used more; (2) they have a unique expertise but should be used in places other than in pharmacies; and (3) they do not really have a role in healthcare—or only a superficial one.

#### 3.1.1. A Unique Expertise?

The special knowledge of the pharmacist was emphasized by some informants, with knowledge about interactions and side effects as some of the examples.

“They understand […] what’s in the pillbox really well.”

(PH, 12) [20].

Many stated that the expertise of the community pharmacist should be used more in the future, especially in counseling and other ways of supporting the patient (note that the word patient was used in this context). In this sense the role of the pharmacist as a healthcare worker—also when working in a community pharmacy—was clear.

“The pharmacist should be given a stronger position. And be more able to use her/his expertise for medicine counseling. […] When it comes to the pharmacies, then it is about the pharmacist’s counseling.”

(PH, 9) [20].

“…the pharmacy personnel have an expertise that we need help and support from.”

(5) [20].

Also, the community pharmacists’ professional willingness to inform the patients about medicines was believed to be important.

“[The patients should] get the information they need in order to take the medicine, and to be able to pose the questions they want.”

(PH, 9) [20].

One opinion among the informants was that having pharmacists in the community pharmacy was important in regard to safety. This includes finding prescription errors potentially harmful to patients, or informing patients about use of medicines in order to prevent incorrect use.

“The reason for having pharmacists in the community pharmacies is to stop possible mistakes made in healthcare from being transferred to the customer.”

(PH, 10) [20].

“Old people have so many strange combinations. You should be able to catch those at the pharmacy.”

(PH, 1) [20].

#### 3.1.2. Pharmacist Counseling Outside the Community Pharmacy

It was sometimes stressed by informants that tasks performed in a community pharmacy need to be done in close collaboration with the rest of the healthcare team, implying that pharmacies are not autonomous healthcare settings.

“It´s important that there are […] pharmacists who know about not only pharmaceuticals, chemistry, and how they work. But who also have some medical knowledge.”

(2) [20].

Some of the informants, however, had the opinion that pharmacists’ expertise and counseling should be used in other healthcare settings (e.g., hospitals, healthcare centers) rather than in pharmacies. For example, they could empower patients in the healthcare sector by increasing patients’ knowledge about medicines:

“Pharmacists who know a lot about medicines […] close to the patients, I think that’s a success factor.”

(PH,12) [20].

Another perspective was that the pharmacists should focus on prescribers, rather than patients, and work together with prescribers in order to, e.g., diminish incorrect prescriptions.

”The pharmacist has a much more in-depth knowledge about medicines than doctors do. […] The pharmacist has a natural place in the healthcare team.”

(2) [20].

#### 3.1.3. Could Perform Easy Tasks, or None at All

One view on the pharmacists’ expertise was that their knowledge was not unique, but instead exchangeable and superficial. According to informants holding this view, the tasks suitable for pharmacists to perform are less complicated, e.g., taking care of healthy individuals. For these informants, the community pharmacies were a place where patients go before going to primary healthcare. Another task mentioned was to merely “help” prescribers in order to free time for them.

One polarized view in regard to community pharmacists as part of healthcare was that they, because of lack of expertise, should not be giving advice at all:

“For us [the organization the informant represented], they don´t exactly have a very good role.”

(8) [20].

#### 3.1.4. Underused Pharmacists

Many informants, especially those who were pharmacist/professional representatives, had the opinion that the pharmacist is underutilized. The reason for this was not discussed much, but it was occasionally stated that the pharmacist’s knowledge has not been promoted enough.

“The pharmacists’ expertise is absolutely not well utilized; […] unfortunately, the pharmacists seem okay with that. They mumble a bit in the wings, but they don’t do anything about it.”

(PH, 1) [20].

### 3.2. Business—A Market with Consumers

A typical view on pharmacies as a market (business perspective) was that a pharmacy is a place where the customer should demand products and services. Also, services often considered as part of healthcare were put in a business perspective. One example was that health tests could be delivered, but only if demanded by customers, but that:

“…it’s possible that there is not so much demand from the customers yet for that type of service.”

(6) [20].

However, some informants also mentioned that this view on tests was problematic because customer-driven testing could be both unnecessary and damaging for the relationship between the patient, the pharmacy, and the rest of the healthcare sector. This was said to be a risk if the testing is made without any consideration of how to interpret the results.

“You take a blood pressure at the pharmacy, and when you go to the community healthcare center because the pharmacy said that you are in a risk zone, the community healthcare center says “they are out of their minds”. It could be that it says so on a paper, theoretically, that this is a risk, but this is okay, and you don’t need to worry.”

(PH, 12) [20].

The business perspective was further underscored by statements such as:

“They [the pharmacy] can’t do that. They can only tell you that you have high blood pressure”

(7) [20].

This indicates that there is no actual healthcare expertise in pharmacies. Also, dispensing was viewed as a business activity only, with the implication that medicine counseling is not a prime interest for pharmacy owners.

Competition in the market was seen by some politicians as the only mechanism for determining which activities pharmacies perform.

“It should not be the politics that have the visions […] This should rather be a market that grows gradually. Where they could see—what can we do in order to meet our customers’ expectations better?”

(4) [20].

It was also stated that the pharmacies should take the lead in this development.

”How do the pharmacies think themselves? […] It is important that they send signals to us politicians that you need to think about how to finance different services, so that we also could be offering these services. Maybe in competition with the primary care centers.”

(3) [20].

No politician explicitly expressed that it is their task to formulate goals for the pharmacies; on the contrary, they said:

“It is not the task of the politics to come up with all the ideas.”

(3) [20].

However, one politician said that the state-owned Apoteket AB should be used by the state, *i.e.*, politicians, to develop the rest of the pharmacy sector.

Referring to the recent liberalization of the Swedish community pharmacy sector, one interviewee from a political party stated that the rationale of “better use of medicines” (which was one of the original rationales for the liberalization) was presented in order not to get *worse* use of medicines after the reform, not because the reform could lead to a better use of medicines. The informant reported a fear that the community pharmacies otherwise could have destroyed the efforts made in order to improve the drug utilization (with, e.g., medicines use reviews), because the pharmacies’ prime interest was believed to be to sell as much medicine as possible:

“The meaning of medicines use reviews […] is to diminish the use of medicines generally. […] And that affects the pharmacies negatively. […] Because they get to sell less.”

(6) [20].

“We didn’t want the pharmacies to be able to influence the efforts made in order to improve drug utilization.”

(6) [20].

Regarding merchandise in pharmacies, the opinions among the informants differed; some viewed it as an important part, while other saw it as detrimental.

“I would feel safe if I need some regular skin care products. That the pharmacy, that is where I should be able to buy products that I know help.”

(PH, 11) [20].

However, interviewees were also negative toward selling too much merchandise in the pharmacies, as this, according to some informants, could not be combined with being a part of the healthcare sector.

“I have some issues with when it gets to be too much business […] with a lot of shampoos.”

(PH, 9) [20].

“If the customer feels that there is no difference between buying Alvedon [paracetamol/acetaminophen product] in a gas station or going to a pharmacy and getting your medicines, then we don’t need any pharmacies.”

(PH, 9) [20].

### 3.3. Distribution

Overall the informants, in particular those from the political and patient organizations, viewed the pharmacies’ most important task to be to distribute (and sell) either only medicines, or medicines and other products. This opinion was also present among informants who were pharmacist/professional representatives.

“The most important task […] is to be a logistician. To give the patient the right package.”

(PH, 12) [20].

It was stated by one interviewee that this is the only thing pharmacies should do, as well as, ideally, delivering to the customers’ homes.

“When I get my grocery store home delivery, well then I´m home to receive it. And then I´ll have to be at home to get my pharmacy delivery also.”

(8) [20].

Many informants had more than one perspective on pharmacies, often including distribution/logistics.

“Pharmacy means two things: first […] it is a part of the healthcare treatment that you have. And secondly, it is to supply medicines.”

(7) [20].

According to one informant, the focus could be on distribution because it is more tangible than some other roles:

”The distribution role/function, that you can see. But the other two [tasks; informant is referring to information and safety], not even the state see them. Not even the county councils, who buy pharmacy services, see it.”

(PH, 10) [20].

## 4. Discussion

In this study of influential stakeholders’ views on community pharmacies/pharmacists, and their role and tasks, it is evident that many of them see pharmacies as being predominantly (retail) businesses, to some degree logistics centers, and less often as part of the healthcare sector.

The results show a great discrepancy regarding the underlying comprehension of some important concepts. The phrases “better use of medicines” as well as that “pharmacies should be a part of the healthcare system” (according to Swedish law, the pharmacists working in the community pharmacies are a part of the healthcare sector.) were interpreted very differently. The political representatives seemed to be on the verge of worrying that community pharmacists might obtain an increased role in medicine use, while the pharmacist/professional representatives hoped for a more prominent role concerning this.

Regarding the different definitions of the phrase “being part of the healthcare sector”, some informants thought that it meant that pharmacists should be used to perform complicated tasks (e.g., for the safety for the patients), whereas others interpreted it as *only* to act as logistics centers. The last is not in line with pharmaceutical care [21], which to a large degree is wished for and driven by pharmacists.

These two discrepancies show the importance of trying to understand the underlying comprehensions of an expression in order to get a meaningful discussion. Otherwise arguments from, e.g., pharmacists will not be taken into consideration in political processes, as they will be understood in a way other than intended.

Pharmacists’ expertise was acknowledged by many, in most cases informants who were pharmacist/professional representatives themselves, and they generally regarded this expertise as specific and not exchangeable. However, they did not have much of a concrete view on how this expertise should be used—either in community pharmacies or elsewhere. In fact, the examples given had a tendency to be technical, e.g., having systems or knowledge to identify drug-drug-interactions—tasks that to a large degree might be performed by other professions, or even by computer programs. The lack of concreteness shown in this study has also been shown in an earlier study on the view of pharmacists in Sweden [22], and is an apparent problem if one hopes for a change towards more use of the pharmacists’ knowledge. Also, a lack of concreteness in general was noted regarding the justification of community pharmacies; no informant expressed that community pharmacies have special characteristics (for example access to the medicines) that make them an ideal place for pharmacists’ counseling. The results show that the informants who were not pharmacist/professional representatives had a more business-oriented view of the community pharmacies. The interviews also indicate that activities such as health tests were considered as primarily business activities. This attitude could be an explanation for why most of the initial rationales for the liberalization of the community pharmacy ownership (e.g., accessibility to pharmacies, efficiency, and price pressure) mainly seemed to be inspired by New Public Management (NPM) [12]. NPM is a collection of ideas, but one prominent characteristic is that competition between organizations increases efficiency [23]. Choices made by consumers in the market are seen, in general, as more effective than decisions made by a few [8], and consumers are seen as the main driving force for the development of services. The question is what kind of services could be demanded, as it is difficult for the customers/patients to have a thorough understanding of what the professional—in this case the pharmacist—could offer [6].

The third perspective (apart from business and healthcare) where pharmacies were seen as distributors of/logistics centers for medicines was distinctly present in the interviews. If this perspective comes to dominate pharmacy sector policies even more, it can possibly have negative consequences for treatment outcomes, as pharmacist counseling has been shown to improve these [24,25].

According to this study, *i.e.*, the views of important and powerful stakeholders, community pharmacies in Sweden are about to develop in a different direction than in many other countries—not towards becoming healthcare providers [5], but rather towards a more business-centered model. This is partly due to the liberalization reform, which was initiated and carried out by politicians, who in this study generally had more of a business perspective with elements of the distribution perspective.

The politicians did not view it as their task to have ideas for, or take the lead in, the development of the community pharmacy sector. However, the results show that pharmacists themselves do not seem to take the lead either. Tendencies in this direction have also been shown in Norway, where there is also a pharmacy market with liberal owner regulations [26]. The consequences of these disengaged attitudes could have a detrimental effect on community pharmacists as healthcare workers, and, in the end, affect patient outcomes negatively.

One limitation of this study is the choice of organizations. Snowballing as a sampling strategy has the disadvantage that only informants in a specific network might be included [27]. In order to diminish this disadvantage, the snowballing technique was combined with strategic sampling. Another limitation is that only one informant was interviewed within each organization. It is thus possible that the views of the informants are their own rather than their organizations’. However, the organizations themselves did choose the informant within each organization.

## 5. Conclusions

This study contributes to the understanding of influential stakeholders’ views on the community pharmacy and the activities performed by the community pharmacist. The views on community pharmacies as more or less just distribution centers seemed to be widespread. The study shows a gap between pharmacist/professional organization representatives, viewing community pharmacy/pharmacists as a part of healthcare, and politicians, generally viewing community pharmacy more as businesses. Different connotations for expressions such as “better drug use” might not be visible on the surface. Hence, it is important to understand the underlying assumptions that different stakeholders have in policy discussions, and for all stakeholders to be more specific about their own. As can be seen from this Swedish example, it could otherwise lead to misunderstandings and wrong expectations on what a reform could bring about. If community pharmacists want something else other than the business or distribution orientations, they will have to be better at understanding the viewpoints of other stakeholders, and be clear about their own. Also, in Sweden, neither politicians nor any other stakeholder seemed to be willing to take the lead in the development of community pharmacies. From a societal or patient perspective, an active development towards using resources, including pharmacists’ expertise, in an optimal way to secure as good an outcome of medicine treatments as possible would be preferred. Influential stakeholders—politicians or others—need to be active in this development.
